# Non-destructive histomorphological identification of Late Pleistocene burned bone fragments using synchrotron radiation X-ray CT at SPring-8

**DOI:** 10.1038/s41598-026-50208-8

**Published:** 2026-05-19

**Authors:** Junmei Sawada, Minoru Yoneda, Kentaro Uesugi, Masato Hoshino, Seiya Watanabe, Naoki Miyamoto, Kazuhiro Uzawa, Takeji Toizumi, Rin Anbo, Fumiko Saeki, Yuzo Yanagita

**Affiliations:** 1 Institute of Physical Anthropology, Niigata University of Health and Welfare, Shimami 1398, Kita-ku, Niigata, 950-3198 Japan; 2 The University Museum, The University of Tokyo, Tokyo, Japan; 3 Japan Synchrotron Radiation Research Institute, Sayo, Hyogo Japan; 4 Forensic Science Laboratory of Hyogo Prefectural Police HQ, Kobe, Hyogo Japan; 5 Faculty of Contemporary Sociology, Shiseikan University, Hagi, Yamaguchi Japan; 6 Pinghu Normal College, Jiaxing University, Jiaxing, China; 7 Organization for the Strategic Coordination of Research and Intellectual Properties, Meiji University, Tokyo, Japan; 8 Sasebo City Board of Education Cultural Properties Division, Sasebo, Nagasaki, Japan

**Keywords:** SR X-ray CT, Taxonomic identification, Burned bone fragments, Bone histomorphology, Late Pleistocene, Terminal Paleolithic, Ecology, Ecology, Evolution, Zoology

## Abstract

Fukui Cave, located in the southwestern Japanese Archipelago, is a cave site containing cultural layers dating from the Late Pleistocene to the early Holocene. This study aimed to identify the animal taxa of burned bone fragments excavated from Layer IV of the cave, dated to approximately 16,000 years ago, using a non-destructive histomorphological approach. Because faunal remains are extremely scarce at Palaeolithic sites in the Japanese Archipelago, these specimens provide important evidence for understanding the relationship between humans and animals during the Late Pleistocene. However, all excavated bones were burned (calcined) fragments less than 1 cm in length, making macroscopic taxonomic identification difficult. To address this, synchrotron radiation X-ray computed tomography (voxel size: 2.74 μm) was performed at SPring-8 to analyze the internal bone microstructure. As a result, secondary osteons were identified in three of the seven burned fragments, and plexiform bone in one. The cross-sectional areas of osteons and Haversian canals were measured and compared statistically with reference data from various mammalian taxa, taking into account possible shrinkage due to burning. The results indicated that these fragments all fall within the range of medium-sized artiodactyls, such as deer or wild boar. In contrast, derivation from large mammals such as Naumann’s elephant or Yabe’s giant deer, which were extinct in the Late Pleistocene, can be excluded. These findings demonstrate the effectiveness of non-destructive histomorphological identification using high-resolution CT for burned bone fragments and provide new insights into animal exploitation by Late Pleistocene humans in the Japanese Archipelago.

## Introduction

### Burned bone fragments from the Late Pleistocene at Fukui Cave

**Fig. 1:**
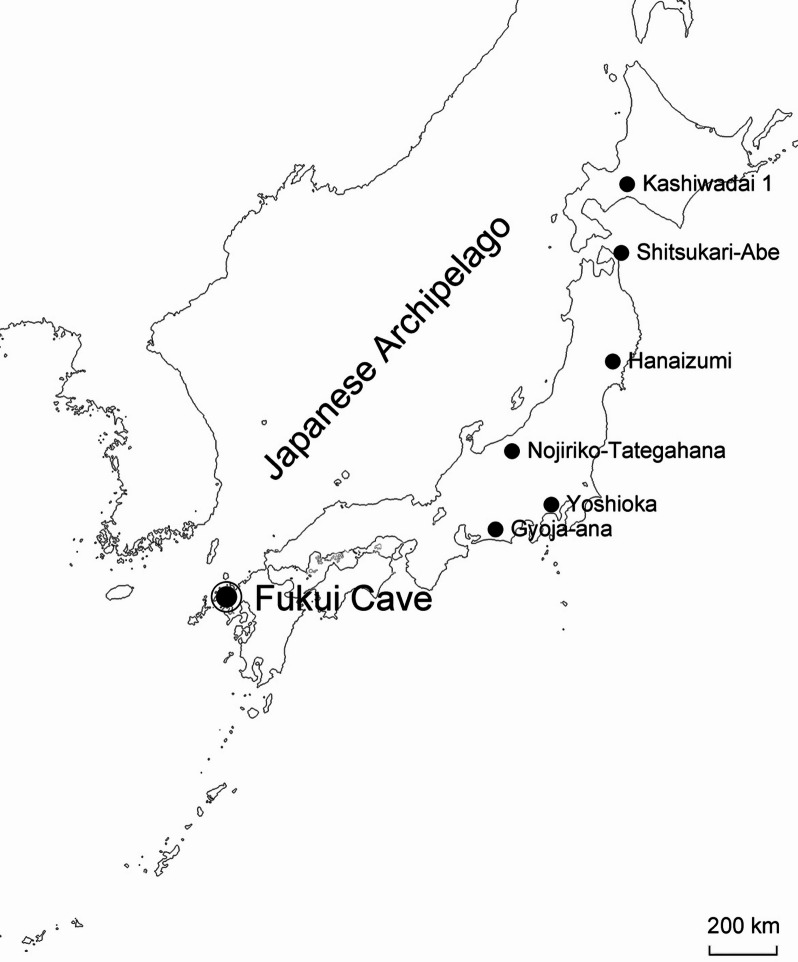
Fukui Cave and Paleolithic sites in the Japanese Archipelago with recovered animal bones.

Fukui Cave (33°17′21″ N, 129°42′36″ E) is located in Sasebo City, Nagasaki Prefecture, in the southwestern part of the Japanese Archipelago (Fig. 1), and has long been recognized as an archaeological site spanning the Late Pleistocene to the earliest Holocene, culturally from the Paleolithic to the emergence of Jomon pottery^1–4^. In recognition of its high archaeological significance, the cave was designated a Special Historic Site by the Agency for Cultural Affairs of the Government of Japan in 2024^5^.

**Fig. 2:**
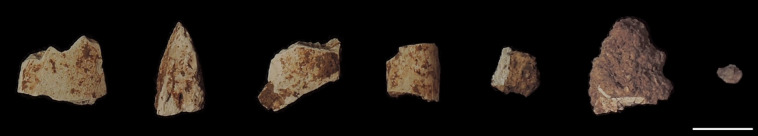
Burned bone fragments from Fukui Cave (left to right: FK-B-001, FK-B-002 … FK-B-007). Scale bar = 5 mm.

Excavations conducted between 2011 and 2015 uncovered a number of burned bone fragments in Layer 4 (Fig. 2), a terminal Paleolithic deposit (ca. 16 ka BP)^6^. The excavated area of Layer 4 measured 1.12 m², and the burned bones were concentrated in a small portion of this space. The curator from the Sasebo City Board of Education who authored the excavation report suggested that a hearth may have existed near the find spot of the burned bones. Based on their size and bone tissue characteristics, the fragments were thought to represent compact bone from terrestrial medium and/or large-sized mammals larger than small mammals such as rodents.

The burned bone fragments exhibited a gray coloration due to burning, with some showed a white coloration. Considering the observed whitening and the inferred exposure to high temperatures (see the Materials section), the materials examined in this study are generally regarded as calcined bone, which is exposed to temperatures above approximately 550°C^7–10^. Only these calcined bone fragments were recovered from this stratigraphic layer, and no unburned bones were found. In general, bone remains were scarce in the excavation of Fukui cave^6^, suggesting that the depositional environment was not favorable for bone preservation. However, exposure to high temperatures likely resulted in the complete combustion of organic components such as collagen and increased the crystallinity of bioapatite^7,8,11–13^, thereby making the bone more resistant to microbial decomposition and chemical dissolution.

It is generally accepted that human groups began to inhabit the Japanese Archipelago in the Late Pleistocene, around 40–35 ka BP^14,15^. The subsequent Paleolithic period, which lasted until approximately 15 ka BP, yielded more than 14,000 sites^16^. However, many of these sites are situated in acidic geological environments derived from volcanic deposits, and, combined with the warm and humid climate that promotes the decomposition of faunal remains, animal bones are only rarely preserved^15,17–19^. In the Japanese Archipelago, only six Paleolithic sites yielded animal remains: the Kashiwadai 1 site (ca. 26–27 ka BP, medium-sized cervids)^18^; Shitsukari-Abe Cave (ca. 20–39 ka BP, hare, Japanese serow, moose, brown bear)^20,21^; the Hanaizumi site (ca. 26 ka BP, bison, Yabe’s giant deer, moose, Naumann’s elephant)^22,23^; and the C-Area of the Yoshioka site group (ca. 25 ka BP, wild boar)^24^, Gyoja-ana cave site (ca. 20 ka BP and 27–28 ka BP, medium-sized mammals)^25,26^, and the Nojiriko-Tategahana lake site (ca. 54–38 ka BP, Naumann’s elephant, Yabe’s giant deer, moose)^27,28^.

Consequently, the actual nature of animal exploitation by Late Pleistocene humans in the Japanese Archipelago remains poorly understood. By the end of the Late Pleistocene, several large mammalian species, including elephants and Yabe’s giant deer, had gone extinct in the archipelago^29–33^. Whether these extinction events were related to human activity during the Paleolithic period remains unresolved^15,19^.

In this context, the burned bone fragments from the terminal Paleolithic of Fukui Cave are of considerable importance for understanding human–animal relationships in the Late Pleistocene of the Japanese Archipelago. Nevertheless, the fragments are heavily degraded, and all measure less than 1 cm in maximum length, making taxonomic identification through macroscopic examination extremely difficult.

### Histomorphological taxonomic identification of small bone fragments

Taxonomic identification of animal bones from archaeological sites represents one of the most fundamental approaches for investigating past human use of animals. Standard methods of taxonomic identification generally rely on macroscopic morphological observation of specimens^34^. However, for highly fragmented bone pieces with little or no remaining macroscopic anatomical features, histomorphological approaches have proven effective^35–40^.

Mammalian bones are fundamentally composed of two types of bone tissue, namely spongy (cancellous) bone and compact (cortical) bone. Compact bone typically contains numerous vascular canals. In many mammals, particularly in the diaphyses of long bones, the compact bone is predominantly composed of osteons (secondary osteons, Haversian systems), which consist of longitudinally oriented canals (Haversian canals) surrounded by concentric lamellae, except in very young individuals. The size of osteons and Haversian canals are influenced by multiple factors, including body mass, lifestyle, and locomotor ability^41,42^, but interspecific differences have long been recognized^43,44^. In non-human animals, osteon banding, in which osteons appear in linear arrangements, is sometimes observed^40,45^. In addition, in certain mammals—particularly artiodactyls such as wild boars, deer, and cattle, perissodactyls such as horses, and carnivores such as dogs and bears—the compact bone of the limbs often exhibits a prominent development of plexiform bone, a distinctive structure in which vascular channels are arranged in a brick-like pattern and surrounded by lamellar bone^46–53^. Although plexiform bone can appear in human infants and children^54–56^, it has not been reported in adult human bone.

Based on accumulated comparative histomorphological knowledge, the presence or absence of osteon banding or plexiform bone, as well as the size of osteons and Haversian canals, provides an effective means of identifying animal taxa. Although the method has inherent limitations in accuracy and applicability, histomorphological analysis has proven reliable for taxonomic identification^40,43,55,57–62^. In the case of highly fragmented burned bones, such as those from Fukui Cave, the application of biochemical identification methods, including DNA analysis or Zooarchaeology by Mass Spectrometry (ZooMS), is generally challenging due to high-temperature burning, which degrades DNA and collagen and prevents molecular identification^63,64^. Under these circumstances, histomorphological approaches currently represent the most practical method for taxonomic identification.

Conventional histomorphological analysis typically requires thin-sectioning of bone fragments and preparation of microscope slides, which entails partial destruction of the specimens. When materials are scarce or when preservation of cultural heritage precludes destructive analysis, non-destructive histomorphological approaches are required. Computed tomography (CT) enables the non-destructive observation of bone microstructure. While medical and industrial CT scanners typically achieve voxel resolutions of several hundred micrometers, osteons in most mammals, including humans, are generally less than 500 μm in diameter, and Haversian canals are even smaller^40,65–69^. Therefore, high-resolution X-ray CT is necessary for the detailed investigation of bone microstructure^70^.

### Objective of this study: non-destructive histomorphological taxonomic identification using high-resolution X-ray CT

In this study, we attempted the histomorphological identification of taxonomic group from Late Pleistocene burned bone fragments from Fukui Cave by non-destructively imaging their bone microstructure using synchrotron radiation X-ray CT (SR X-ray CT) at SPring-8, a large synchrotron radiation facility. SPring-8, located in Sayo Town, Hyogo Prefecture, Japan, is capable of generating world-leading synchrotron radiation and operates as a joint-use facility open to researchers from academia and industry both domestically and internationally. Using the CT at this facility, we obtained high-resolution images of the bone microstructure of the Fukui Cave fragments at a voxel size of 2.74 μm.

As prior studies utilizing CT images for histomorphological taxonomic identification of archaeological bones, Bradfield^71^ identified the raw materials of worked bone artifacts from the Middle Stone Age in South Africa, and in forensic science, Andronowski et al.^72^ examined methods for histomorphological differentiation between human and bear metacarpals. However, such studies remain scarce, and no research has yet applied this approach to tiny fragments of burned bone, such as those from Fukui Cave. Establishing this method as a practical and reliable tool requires the accumulation of empirical results. This paper demonstrates that high-resolution X-ray CT is an effective approach for non-destructive taxonomic identification of highly fragmented burned bone. Furthermore, based on the identification results obtained in this study, we aim to provide new insights into the exploitation of animals by Late Pleistocene humans in the Japanese Archipelago.

## Results

### CT observations of the burned bone fragments from Fukui Cave

**Fig. 3:**
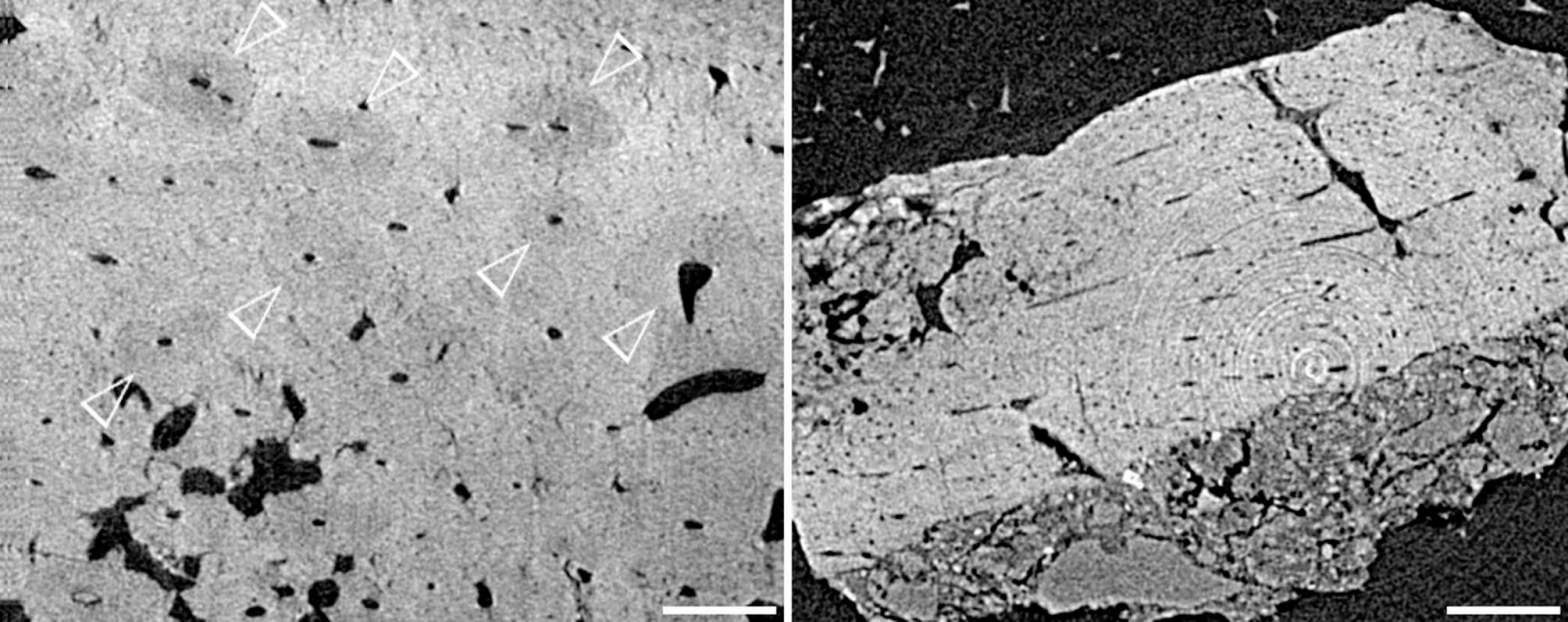
CT images of burned bones from Fukui Cave. Left: osteons in specimen FK-B-003; right: plexiform bone structure in specimen FK-B-007. Arrows point to osteons. Scale bar = 200 μm.

Among the seven burned bone fragments (Specimen IDs: FK-B-001 to FK-B-007) examined by CT, specimens FK-B-002, FK-B-003, and FK-B-006 showed osteons (secondary osteons) distributed across the observable compact bone, constituting its predominant component (Fig. 3). Haversian canals were clearly visible at the centers of osteons, but Haversian lamellae and lacunae could not be observed because they were beyond the resolution limits of the CT. FK-B-007 exhibited plexiform bone throughout the observable area (Fig. 3). No pathological features, such as abnormally enlarged canals indicative of osteoporosis, were observed in FK-B-002, FK-B-003, FK-B-006, or FK-B-007, suggesting that all retained normal histological characteristics. FK-B-001, FK-B-004, and FK-B-005 displayed structures resembling Haversian canals, but the outlines of osteons were indistinct, making it difficult to clearly identify bone microstructures.

### Histomorphometric values

Among the calcined bone fragments from Fukui Cave, the cross-sectional area of complete osteons (On.Ar) and the cross-sectional area of the Haversian canal within each osteon (H.Ar) were measured for FK-B-002, FK-B-003, and FK-B-006, in which osteons were identified. Taking into account the estimated burning temperatures of 500–700 °C, Table 1 presents the values under two scenarios: (1) no shrinkage at 500–600 °C (corresponding to the lower bound in the Methods section), and (2) shrinkage at 700 °C (corresponding to the upper bound).

**Table 1:** On.Ar and H.Ar of burned bone fragments from Fukui Cave.

Specimen ID	*N*.On	On.Ar (µm²)				H.Ar (µm²)			
		Lower bound		Upper bound		Lower bound		Upper bound	
		Mean	SD	Mean	SD	Mean	SD	Mean	SD
FK-B-002	3	13743.9	2766.0	18325.2	3687.9	490.5	190.6	654.0	254.1
FK-B-003	21	18174.1	5226.5	24232.1	6968.7	478.7	186.2	638.3	248.3
FK-B-006	6	15936.1	3689.1	21248.2	4918.8	500.5	370.7	667.3	494.2

N.On: Number of osteons

The On.Ar ranges (lower bound–upper bound) were as follows: FK-B-002, 13,743.9 (± 2,766.0) to 18,325.2 (± 3,687.9) µm²; FK-B-003, 18,174.1 (± 5,226.5) to 24,232.1 (± 6,968.7) µm²; and FK-B-006, 15,936.1 (± 3,689.1) to 21,248.2 (± 4,918.8) µm². The H.Ar ranges were: FK-B-002, 490.5 (± 190.6) to 654.0 (± 254.1) µm²; FK-B-003, 478.7 (± 186.2) to 638.3 (± 248.3) µm²; and FK-B-006, 500.5 (± 370.7) to 667.3 (± 494.2) µm² (values represent mean ± SD). The actual On.Ar and H.Ar values are assumed to lie within these ranges.

**Fig. 4:**
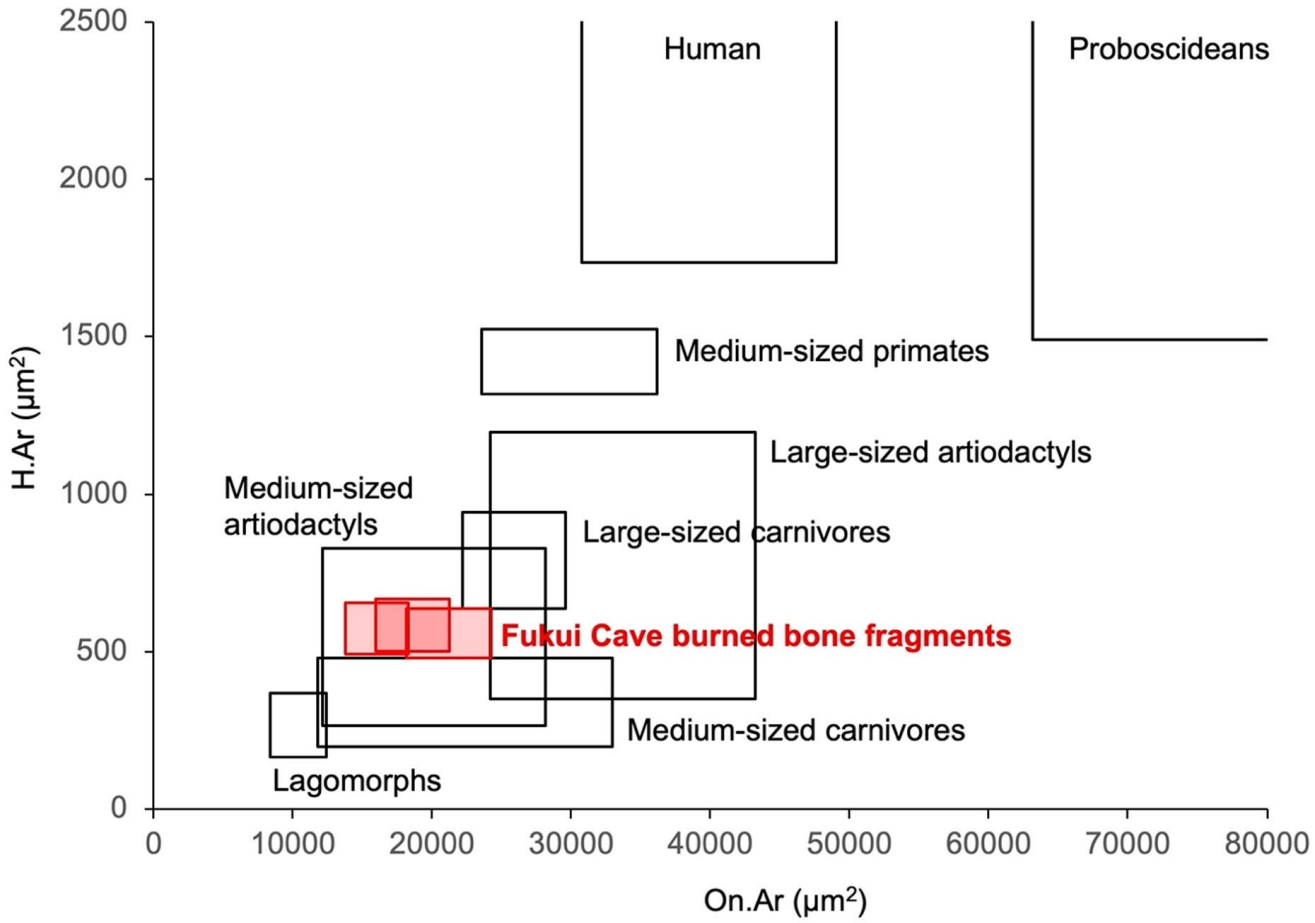
Ranges of mean On.Ar and H.Ar values for the Fukui Cave specimens and comparative mammalian groups, based on histomorphometric data in Tables 1 and 2. The Fukui Cave specimens are, from left to right, FK-B-002, FK-B-006, and FK-B-003. The lower-left corners of the Fukui Cave range indicate values estimated for burning temperatures of 500–600 °C, and the upper-right corners indicate those estimated for 700 °C.

The mean values and standard errors of On.Ar and H.Ar for the comparative faunal specimens are presented in Table 2. Since animals belonging to the same order and of similar body size tended to exhibit comparable On.Ar and H.Ar values, the comparative specimens were grouped according to order-level taxonomy and body size: proboscideans (Naumann’s elephant, fossil elephant, Asian elephant), human, medium-sized primates (macaque), lagomorphs (leporid), medium-sized artiodactyls (wild boar, sika deer, ancient sika deer, reindeer, Japanese serow), large-sized artiodactyls (Yabe’s giant deer, cattle, bison), medium-sized carnivores (Japanese marten, dog, raccoon dog, red fox, leopard), and large-sized carnivores (brown bear). A bivariate plot was then generated with On.Ar on the X-axis and H.Ar on the Y-axis, in which the ranges of each animal group were represented by rectangles defined by the minimum and maximum mean values of On.Ar and H.Ar calculated across individual specimens within each group (Fig. 4).

**Table 2:** Histomorphology of comparative specimens.

Taxon	Specimen ID	Tissue	On.Ar (µm²)			H.Ar (µm²)		
			*N*	Mean	SE	*N*	Mean	SE
Naumann’s elephant	PLN01Tp	SO	18	224893.3	27974.9	18	5584.1	987.6
Pleistocene elephant fossil	EKZ97Fa	SO	10	175852.0	23663.3	10	3711.8	656.2
Asian elephant	Ele01Fp	SO	52	63151.7	2045.0	52	1490.6	76.8
Human	Homo01Ha	SO	30	30722.8	2488.9	30	1975.1	202.4
Human	Homo03Ha	SO	39	31463.8	1687.9	39	2040.9	143.3
Human	Homo01Fa	SO	54	41846.8	2177.3	54	2524.5	161.9
Human	Homo02Fa	SO	55	33931.6	1648.9	55	1734.3	134.1
Human	Homo04Fa	SO	46	35774.2	1650.4	46	2336.2	135.7
Human	Homo05Fa	SO	22	36560.1	2572.6	22	1748.7	210.0
Human	Homo06Fa	SO	26	49038.7	4502.8	26	2334.5	260.4
Human	HomoPf01	SO	1078	35988.9	497.2	1078	1938.0	41.8
Human	HomoPf02	SO	996	44532.9	711.2	996	3667.4	123.6
Human	HomoPf03	SO	1023	35057.4	533.0	1023	3207.2	59.9
Macaque	Mac01Fa	SO	12	36154.1	3087.3	12	1317.3	174.7
Macaque	MacHa01	SO	-	23984.0	(SD: 6324.2)	-	1442.1	(SD: 469.0)
Macaque	MacHa02	SO	-	23583.4	(SD: 4740.2)	-	1523.2	(SD: 531.9)
Leporid	Lep01Hp	SO	6	10869.5	773.8	6	168.5	31.1
Leporid	Lep01Fl	SO	13	11136.7	1292.2	13	165.8	23.3
Rabbit	RabMa	SO	50	8340.0	460.3	50	367.5	32.5
Wild boar	Sus01Ha	SO	87	19673.2	767.1	87	646.0	30.8
Wild boar	Sus02Ha	SO	28	25294.5	2087.0	28	463.7	31.5
Wild boar	Sus01Fa	SO	29	25740.1	1403.7	29	827.7	66.8
Wild boar	Sus02Fa	SO	11	24932.9	3243.8	11	680.7	116.6
Sika deer	Cer02Ha	SO, PF	51	19076.6	872.5	51	469.4	19.5
Sika deer	Cer03Ha	SO, PF	30	20618.6	1286.8	30	503.5	31.6
Sika deer	Cer01Ra	SO	49	12144.2	620.6	49	263.6	13.4
Sika deer	Cer01Fa	PF						
Sika deer	Cer05Fa	PF						
Sika deer	Cer01Fp	SO	39	17965.2	1412.5	39	506.8	35.4
Sika deer	Cer02Fp	SO	58	28138.8	1272.2	58	575.0	24.7
Sika deer	Cer03Fp	SO	48	22522.9	1407.0	48	722.2	45.8
Sika deer	Cer01Ta	SO, PF	66	12920.0	505.7	66	408.9	20.9
Sika deer	Cer04Ta	PF						
Ancient sika deer	CPR91Fp	SO	37	23922.7	2068.4	37	459.0	40.7
Ancient sika deer	CPR92Ta	SO	117	15037.8	492.2	117	326.5	13.8
Reindeer	Ran01Fp	SO	40	17072.0	1080.2	40	272.2	19.5
Japanese serow	Cap01Fa	SO	32	26140.1	1461.8	32	485.8	36.8
Yabe’s giant deer	SMY01M	SO	24	40086.5	3212.8	24	350.0	53.3
Yabe’s giant deer	SMY02Ra	PF						
Cattle	Bos01Ha	PF						
Cattle	Bos04Ha	SO	71	43209.7	1740.1	71	591.4	28.7
Cattle	Bos05Ha	SO	30	25216.9	1603.3	30	512.1	22.5
Cattle	Bos01Ra	SO, PF	13	29406.6	1959.2	13	671.8	50.7
Cattle	Bos02Fa	SO	25	31532.4	2000.3	25	699.6	56.6
Cattle	Bos03Fa	SO	16	28061.3	2700.5	16	714.8	84.5
Cattle	BosMa	SO	160	31725.4	889.2	160	1196.7	51.1
Bison	Bis01Fp	SO	88	24199.5	1039.4	88	770.3	28.8
Japanese marten	Mar01Ha	SO	10	11766.2	1438.9	10	197.5	35.3
Dog	Can01Fa	SO	26	12789.2	1017.4	26	298.2	32.0
Dog	Can01Ta	SO	65	15291.4	609.8	65	273.3	16.3
Raccoon dog	Nyc01Fa	PF						
Raccoon dog	Nyc01Fp	SO	13	22022.3	3202.3	13	480.1	53.5
Red fox	Vul01M	SO	21	32937.9	2345.5	21	294.6	31.0
Leopard	Pan01Fa	SO	26	29351.5	4010.7	26	772.3	68.3
Brown bear	Urs01Ha	SO	114	26356.1	1049.6	114	719.5	27.8
Brown bear	Urs01Ra	SO	109	29614.9	1109.3	109	942.1	38.3
Brown bear	Urs01Fa	SO	131	22203.2	722.9	131	637.3	27.8
Brown bear	Urs01Ta	SO	138	22496.6	694.5	138	715.9	30.3

N.B: Number of bones; SO: Secondary osteons; PF: Plexiform bone

When the ranges from the lower to upper bounds of On.Ar and H.Ar for FK-B-002, FK-B-003, and FK-B-006 from Fukui Cave were indicated on the graph as rectangles, all three specimens fell within the range of medium-sized artiodactyls, and were next close to the ranges of carnivores. In contrast, the values for humans and proboscideans were clearly distant from those of the Fukui Cave burned bones. Among the large artiodactyls, the values for bovids (cattle and bison) were relatively close to the Fukui Cave range, whereas Yabe’s giant deer, which became extinct in the Pleistocene, lay farther away (Table 2; Fig. 4).

### Statistical results

Assuming that no shrinkage of the bone tissue structure occurred at a burning temperature of 500–600 °C, significant differences (*p* < 0.05), determined by Games–Howell tests, in either or both On.Ar and H.Ar were detected between FK-B-002, FK-B-003, and FK-B-006, and the following comparative specimens: Naumann’s elephant, fossil elephant, Asian elephant, human, macaque, sika deer (one of seven specimens), Yabe’s giant deer, cattle (three of five specimens), red fox, and brown bear (one of four specimens) (Table 3). Additional significant differences were also observed between each of FK-B-003 and FK-B-006 and Japanese serow and brown bear (one of the remaining three specimens), as well as between FK-B-003 and leporid, wild boar (one of four specimens), sika deer (one of the remaining six specimens), bison, Japanese marten, dog (one of two specimens), and brown bear (one of the remaining two specimens) (Table 3).

**Table 3:** P values from the Games–Howell test for pairwise comparisons between the histomorphometric values of the Fukui Cave specimens (assuming no shrinkage by burning) and those of comparative mammal specimens.

Taxon	Specimen ID	On.Ar			H.Ar		
		FK-B-002	FK-B-003	FK-B-006	FK-B-002	FK-B-003	FK-B-006
Naumann’s elephant	PLN01Tp	**< 0.001**	**< 0.001**	**< 0.001**	**0.023**	**0.022**	**0.024**
Pleistocene elephant fossil	EKZ97Fa	**0.011**	**0.013**	**0.012**	0.088	0.086	0.091
Asian elephant	Ele01Fp	**< 0.001**	**< 0.001**	**< 0.001**	**0.045**	**< 0.001**	**0.041**
Human	Homo01Ha	**0.008**	**0.022**	**0.009**	**< 0.001**	**< 0.001**	**0.002**
Human	Homo03Ha	**0.006**	**< 0.001**	**< 0.001**	**< 0.001**	**< 0.001**	**< 0.001**
Human	Homo01Fa	**< 0.001**	**< 0.001**	**< 0.001**	**< 0.001**	**< 0.001**	**< 0.001**
Human	Homo02Fa	**0.003**	**< 0.001**	**< 0.001**	**0.003**	**< 0.001**	**0.006**
Human	Homo04Fa	**0.001**	**< 0.001**	**< 0.001**	**< 0.001**	**< 0.001**	**< 0.001**
Human	Homo05Fa	**< 0.001**	**< 0.001**	**< 0.001**	**0.014**	**0.002**	**0.027**
Human	Homo06Fa	**< 0.001**	**< 0.001**	**< 0.001**	**< 0.001**	**< 0.001**	**< 0.001**
Macaque	Mac01Fa	**0.006**	**0.020**	**0.009**	0.205	0.080	0.333
Leporid	Lep01Hp	0.978	**0.009**	0.613	0.695	**0.002**	0.913
Leporid	Lep01Fl	0.999	0.102	0.875	0.679	**< 0.001**	0.902
Wild boar	Sus01Ha	0.541	1.000	0.917	0.991	0.397	1.000
Wild boar	Sus02Ha	0.119	0.598	0.246	1.000	1.000	1.000
Wild boar	Sus01Fa	0.077	0.055	0.052	0.752	**0.028**	0.961
Wild boar	Sus02Fa	0.555	0.980	0.842	1.000	0.998	1.000
Sika deer	Cer02Ha	0.653	1.000	0.988	1.000	1.000	1.000
Sika deer	Cer03Ha	0.493	1.000	0.899	1.000	1.000	1.000
Sika deer	Cer01Ra	1.000	**0.025**	0.875	0.888	**0.015**	0.997
Sika deer	Cer01Fp	0.955	1.000	1.000	1.000	1.000	1.000
Sika deer	Cer02Fp	**0.037**	**< 0.001**	**0.006**	1.000	0.991	1.000
Sika deer	Cer03Fp	0.261	0.937	0.491	0.924	0.084	0.999
Sika deer	Cer01Ta	1.000	0.077	0.968	1.000	1.000	1.000
Ancient sika deer	CPR91Fp	0.226	0.923	0.517	1.000	1.000	1.000
Ancient sika deer	CPR92Ta	1.000	0.868	1.000	0.980	0.286	1.000
Reindeer	Ran01Fp	0.977	1.000	1.000	0.910	**0.032**	0.996
Japanese serow	Cap01Fa	0.065	**0.039**	**0.040**	1.000	1.000	1.000
Yabe’s giant deer	SMY01M	**< 0.001**	**< 0.001**	**< 0.001**	0.999	0.996	1.000
Cattle	Bos05Ha	0.093	0.229	0.107	1.000	1.000	1.000
Cattle	Bos04Ha	**< 0.001**	**< 0.001**	**< 0.001**	1.000	0.963	1.000
Cattle	Bos01Ra	**0.021**	**0.025**	**0.014**	0.988	0.614	1.000
Cattle	Bos02Fa	**0.005**	**< 0.001**	**0.001**	0.972	0.475	1.000
Cattle	Bos03Fa	0.079	0.385	0.169	0.989	0.862	1.000
Bison	Bis01Fp	0.146	0.103	0.114	0.790	**< 0.001**	0.981
Japanese marten	Mar01Ha	1.000	0.328	0.976	0.770	**0.008**	0.956
Dog	Can01Fa	1.000	0.262	0.994	0.962	0.283	0.999
Dog	Can01Ta	1.000	0.964	1.000	0.909	**0.028**	0.996
Raccoon dog	Nyc01Fp	0.914	1.000	0.998	1.000	1.000	1.000
Red fox	Vul01M	**0.003**	**0.002**	**0.001**	0.957	0.234	0.999
Leopard	Pan01Fa	0.254	0.793	0.509	0.887	0.191	0.994
Brown bear	Urs01Ha	0.079	**0.001**	**0.025**	0.899	**0.009**	0.998
Brown bear	Urs01Ra	**0.032**	**< 0.001**	**0.003**	0.445	**< 0.001**	0.683
Brown bear	Urs01Fa	0.273	0.605	0.328	0.994	0.441	1.000
Brown bear	Urs01Ta	0.255	0.435	0.275	0.909	**0.014**	0.998

Assuming that shrinkage of the bone tissue structure occurred at a burning temperature of 700 °C, significant differences (*p* < 0.05) in either or both On.Ar and H.Ar were detected between FK-B-002, FK-B-003, and FK-B-006, and the following comparative specimens: Naumann’s elephant, fossil elephant, Asian elephant, human (five of seven specimens), Yabe’s giant deer, and cattle (one of five specimens) (Table 4). Additional significant differences were also observed between each of FK-B-002 and FK-B-003 and human (one of the remaining two specimens), as well as between FK-B-003 and human (one of the remaining specimens), leporid, sika deer (two of seven specimens), ancient sika deer (one of two specimens), reindeer, Japanese marten, dog, red fox, and brown bear (one of four specimens) (Table 4).

**Table 4:** P values from the Games–Howell test for pairwise comparisons between the histomorphometric values of the Fukui Cave specimens (assuming shrinkage by burning) and those of comparative mammal specimens.

Taxon	Specimen ID	On.Ar			H.Ar		
		FK-B-002	FK-B-003	FK-B-006	FK-B-002	FK-B-003	FK-B-006
Naumann’s elephant	PLN01Tp	**< 0.001**	**< 0.001**	**< 0.001**	**0.032**	**0.030**	**0.033**
Pleistocene elephant fossil	EKZ97Fa	**0.013**	**0.017**	**0.015**	0.120	0.113	0.128
Asian elephant	Ele01Fp	**< 0.001**	**< 0.001**	**< 0.001**	0.227	**< 0.001**	0.328
Human	Homo01Ha	0.285	0.971	0.620	**0.028**	**< 0.001**	0.063
Human	Homo03Ha	0.170	0.459	0.215	**0.021**	**< 0.001**	**0.029**
Human	Homo01Fa	**0.007**	**< 0.001**	**< 0.001**	**0.002**	**< 0.001**	**0.002**
Human	Homo02Fa	0.090	**0.031**	0.053	0.080	**< 0.001**	0.137
Human	Homo04Fa	0.058	**0.002**	**0.018**	**0.008**	**< 0.001**	**0.007**
Human	Homo05Fa	**0.039**	0.081	**0.038**	0.130	**0.013**	0.251
Human	Homo06Fa	**0.001**	**0.006**	**0.002**	**0.007**	**< 0.001**	**0.016**
Macaque	Mac01Fa	0.085	0.356	0.158	0.650	0.278	0.868
Leporid	Lep01Hp	0.569	**< 0.001**	0.139	0.594	**< 0.001**	0.823
Leporid	Lep01Fl	0.665	**< 0.001**	0.179	0.586	**< 0.001**	0.814
Wild boar	Sus01Ha	1.000	0.797	1.000	1.000	1.000	1.000
Wild boar	Sus02Ha	0.875	1.000	1.000	0.995	0.735	1.000
Wild boar	Sus01Fa	0.657	1.000	0.989	1.000	0.976	1.000
Wild boar	Sus02Fa	0.995	1.000	1.000	1.000	1.000	1.000
Sika deer	Cer02Ha	1.000	0.635	1.000	0.995	0.639	1.000
Sika deer	Cer03Ha	1.000	0.999	1.000	1.000	0.977	1.000
Sika deer	Cer01Ra	0.703	**< 0.001**	0.224	0.740	**< 0.001**	0.943
Sika deer	Cer01Fp	1.000	0.579	1.000	1.000	0.990	1.000
Sika deer	Cer02Fp	0.374	0.995	0.654	1.000	1.000	1.000
Sika deer	Cer03Fp	0.984	1.000	1.000	1.000	1.000	1.000
Sika deer	Cer01Ta	0.787	**< 0.001**	0.296	0.961	0.138	0.999
Ancient sika deer	CPR91Fp	0.975	1.000	1.000	0.995	0.821	1.000
Ancient sika deer	CPR92Ta	0.980	**0.003**	0.615	0.847	**0.005**	0.985
Reindeer	Ran01Fp	1.000	0.140	0.984	0.758	**< 0.001**	0.953
Japanese serow	Cap01Fa	0.615	1.000	0.977	0.999	0.946	1.000
Yabe’s giant deer	SMY01M	**0.013**	**0.038**	**0.015**	0.920	0.152	0.996
Cattle	Bos05Ha	0.771	1.000	0.999	1.000	0.974	1.000
Cattle	Bos04Ha	**0.010**	**< 0.001**	**< 0.001**	1.000	1.000	1.000
Cattle	Bos01Ra	0.333	0.981	0.650	1.000	1.000	1.000
Cattle	Bos02Fa	0.176	0.660	0.286	1.000	1.000	1.000
Cattle	Bos03Fa	0.687	1.000	0.982	1.000	1.000	1.000
Bison	Bis01Fp	0.791	1.000	1.000	1.000	0.977	1.000
Japanese marten	Mar01Ha	0.777	**0.002**	0.268	0.643	**< 0.001**	0.872
Dog	Can01Fa	0.829	**< 0.001**	0.322	0.813	**0.003**	0.975
Dog	Can01Ta	0.991	**0.005**	0.679	0.758	**< 0.001**	0.953
Raccoon dog	Nyc01Fp	1.000	1.000	1.000	0.999	0.984	1.000
Red fox	Vul01M	0.128	0.517	0.215	0.805	**0.003**	0.972
Leopard	Pan01Fa	0.895	1.000	0.998	1.000	1.000	1.000
Brown bear	Urs01Ha	0.530	1.000	0.906	1.000	1.000	1.000
Brown bear	Urs01Ra	0.269	0.692	0.347	0.924	**0.021**	0.999
Brown bear	Urs01Fa	0.958	1.000	1.000	1.000	1.000	1.000
Brown bear	Urs01Ta	0.934	1.000	1.000	1.000	1.000	1.000

## Discussion

### Histomorphological features of the burned bone fragments observed in CT images and identification of animal taxa

The fact that osteons, Haversian canals, and plexiform bone structures could be observed in CT images of the burned bone fragments from Fukui Cave provides evidence that high-resolution X-ray CT allows for histomorphological identification of animal taxa without destructive sampling, even in small Pleistocene calcined bone fragments. However, in three of the seven specimens, the histological structures could not be clearly observed. In general, bones recovered from archaeological sites are likely to have been subjected to various biological, chemical, and physical damage during long-term deposition in the sediment^34,73,74^; such taphonomic alterations were probably imposed on the present specimens as well.

The On.Ar and H.Ar values of the burned bone fragments fell within the range observed in medium-sized artiodactyls such as wild boar and sika deer (Fig. 4). Therefore, these burned bone fragments were most plausibly derived from a medium-sized artiodactyl. The presence of plexiform bone identified in FK-B-007, a structure characteristic of artiodactyls, also supports this inference. However, the On.Ar and H.Ar values of wild boar, sika deer, and Japanese serow largely overlap (Table 2), making it impossible to identify the species within the medium-sized artiodactyl group based on this histomorphological method alone. Moreover, since the values are also close to those of some carnivores, the possibility that the specimens belong to these taxa cannot be completely ruled out.

Both On.Ar and H.Ar are known to vary within a single bone depending on the sampled region^67,75^, and a fragment does not always reflect the overall histomorphological characteristics of the entire bone^76,77^. Therefore, taxonomic identification based solely on histomorphometric observations requires caution. Even so, the measured values of the burned bone fragments differed markedly from those of proboscideans, Yabe’s giant deer, and humans (Tables 3 and 4), suggesting that the Fukui Cave specimens can be effectively excluded from these taxa.

Taken together, the present analysis indicates that (1) the burned bone fragments from Fukui Cave were most likely derived from a medium-sized artiodactyl, although the possibility of their origin from a carnivore cannot be ruled out; and (2) the possibility that they originated from proboscideans, Yabe’s giant deer, or humans can be considered highly unlikely.

### Utilization of animal resources in the Japanese Archipelago during the terminal Palaeolithic

The mammalian fauna of the Japanese archipelago during the Late Pleistocene included species that still inhabit the archipelago today—such as medium-sized artiodactyls (wild boar, sika deer, and serow) and carnivores (bears, raccoon dogs, and foxes)—as well as extinct taxa including proboscideans (Naumann’s elephant and mammoths) and large artiodactyls (Yabe’s giant deer and bison). Because Palaeolithic sites yielding animal bones were long considered extremely rare in Japan, the limited number of finds led to the widespread image that humans of the time hunted large mammals such as Naumann’s elephant and Yabe’s giant deer^28,78^. This “big-game hunting” view has been disseminated through school textbooks and has become part of the general public’s understanding of the Palaeolithic period.

In recent years, several Palaeolithic sites have yielded faunal remains predominantly consisting of medium- to small-sized mammals such as cervids and hares^18,21^. In addition, analyses of lithic assemblages and studies on pitfall hunting have suggested that medium- and small-sized mammals were among the main hunting targets during the Palaeolithic period^79,80^, and thus a reconsideration of the traditional view that emphasizes the hunting of large game has been proposed^19,81^.

Regarding faunal transitions in the Late Pleistocene of the Japanese Archipelago, Naumann’s elephant and Yabe’s giant deer have traditionally been thought to have become extinct between ca. 20 and 10 ka BP^30,32^. However, Takahashi^82^ and Iwase et al.^29^, who compiled reliable AMS radiocarbon dates for large mammal fossils, demonstrated that no definitive ages later than ca. 25 ka BP are available for Naumann’s elephant and Yabe’s giant deer, and suggested that environmental changes associated with the onset of the Last Glacial Maximum (LGM) around 30 ka BP may have led to their extinction by approximately 25 ka BP. Furthermore, Sawada and Yoshinaga^83^, who reviewed major sites yielding faunal remains from the Incipient Jomon period (ca. 15–12 ka BP), confirmed that the taxonomic composition of the faunal assemblages from this period was broadly similar to that of the succeeding Jomon period (ca. 12–3 ka BP), indicating that large mammals had already disappeared by that time.

However, between ca. 25 and 15 ka BP, reliable AMS radiocarbon dates are extremely scarce not only for Naumann’s elephant and Yabe’s giant deer but also for medium- and small-sized mammals. In other words, the faunal composition of this period itself remains poorly understood, and to examine the faunal transition—including the extinction of large mammals—it is essential to accumulate additional faunal remains from this timespan.

Burned bone fragments from Fukui Cave, dated to approximately 16 ka BP, are important in filling this chronological gap. Although detailed taxonomic identification was not achieved in this study, the specimens were shown to possess morphological characteristics consistent with medium-sized artiodactyls and not with proboscideans or Yabe’s giant deer. This finding provides valuable clues for understanding both the extinction process of large mammals in the Japanese Archipelago and the nature of animal exploitation during the terminal phase of the Palaeolithic period.

## Conclusions

The extinction of large mammals worldwide during the Late Pleistocene has generally been attributed to environmental changes associated with climatic fluctuations and human hunting activities. The relative contribution of these factors differed among regions^84,85^; hence, elucidating local processes in detail is crucial for reconstructing Quaternary natural history and understanding how humans first became involved in animal extinctions. While considerable progress has been made in studies addressing this issue in Europe, Siberia, Australia, and the Americas^86–91^, investigations of the relationship between human activities and faunal changes during the Late Pleistocene have lagged in the Japanese Archipelago, located at the easternmost end of Eurasia.

In this study, burned bone fragments from the terminal Palaeolithic layers of Fukui Cave were analyzed using high-resolution X-ray CT to investigate their histomorphological characteristics. The results indicated that these remains did not derive from large mammals that became extinct during the Late Pleistocene. Although the sample size was limited and the specimens may not represent the overall pattern of animal exploitation at the site, the findings contribute to understanding the extinction processes of large mammals in the Japanese Archipelago. The accumulation of such data is particularly significant in Japan, where faunal remains from Pleistocene human sites are extremely scarce.

Bone histomorphological identification still involves several methodological challenges, such as limited taxonomic resolution and insufficient comparative data by species and skeletal element. Nevertheless, in regions such as the Japanese Archipelago—where bone preservation at Pleistocene archaeological sites is generally poor—methods capable of extracting information from highly fragmented specimens are especially valuable. Non-destructive taxonomic identification based on histomorphological observation using high-resolution X-ray CT is promising, as it allows for the preservation and future utilization of materials and is expected to play an important role in subsequent research.

## Materials and methods

### Burned bone fragments from Fukui Cave

Seven small burned bone fragments (Specimen IDs: FK-B-001 to FK-B-007), presumed to be mammalian cortical bone, recovered from Layer 4 of Fukui Cave (ca. 16 ka BP), were subjected to CT scanning (Fig. 2; Table 5). These specimens are curated by the Board of Education of Sasebo City, where Fukui Cave is located, and the present study was conducted with the Board’s permission.

**Table 5:** Burned bone fragments from Fukui Cave.

Specimen ID	Length	Weight	Bone type	Histomorphological condition	Structure
FK-B-001	7 mm	< 0.1 g	Compact bone	Not good	Osteon?
FK-B-002	7 mm	< 0.1 g	Compact bone	Good	Osteon
FK-B-003	8 mm	< 0.1 g	Compact bone	Good	Osteon
FK-B-004	5 mm	< 0.1 g	Compact bone	Not good	Osteon?
FK-B-005	4 mm	< 0.1 g	Compact bone	Not good	Osteon?
FK-B-006	8 mm	< 0.1 g	Compact bone	Good	Osteon
FK-B-007	2 mm	< 0.1 g	Compact bone	Good	Plexiform bone

These fragments exhibited gray to white coloration due to burning. The color of bone changes with burning: low temperature burning results in a brown coloration, which turns black and then bluish or gray as the temperature rises, and eventually becomes white calcined bone^7,8,92–95^. The relationship between bone color and burning temperature varies with factors such as oxygen availability, combustion duration, and cortical bone thickness; therefore, when estimating burning temperature from color, it is necessary to consider these factors and infer an approximate temperature range. Absolonová et al.^96^ summarized many previous studies on the relationship between burning temperature and bone coloration, reporting that bones become gray at burning temperatures of 500–700 °C. Accordingly, the burned bone fragments recovered from Fukui Cave are considered to have been burned within this temperature range.

Additional analytical methods such as Fourier transform infrared spectroscopy (FTIR), Raman spectroscopy, and X-ray diffraction (XRD) could provide further information on burning temperatures^7,10,11^. However, these analyses were not performed because the specimens are extremely small and fragile, and destructive damage was a concern.

### Synchrotron radiation X-ray CT imaging

SR X-ray CT imaging of the burned bone fragments excavated from Fukui Cave was performed at beamline BL20B2 of the large synchrotron radiation facility SPring-8. The imaging was conducted using monochromatic synchrotron radiation, with the X-ray energy set to 35 keV. Each specimen was mounted on a rotation stage with a custom holder and rotated 180° during image acquisition. The exposure time was 120 msec per projection, and the specimen -to-detector distance was set to 150 mm. A total of 1800 projections were acquired and reconstructed using the filtered back-projection (FBP) method. The resulting three-dimensional dataset had an isotropic voxel size of 2.74 μm. This resolution is considered sufficient for measuring the areas of osteons (approximately 10,000–30,000 μm²) and Haversian canals (approximately 200–1,200 μm²) in the burned bone fragments from Fukui Cave (see the Results section). The reconstructed images were converted to TIFF format and subsequently analyzed using ImageJ.

### Observation and measurement of bone microstructure

For the seven burned bone fragments from Fukui Cave, osteons (secondary osteons) and plexiform bone were examined in the CT images. For fragments in which osteons were identifiable, the cross-sectional area of complete osteons (On.Ar) and the cross-sectional area of the Haversian canal within each osteon (H.Ar) were measured. Measurements were performed on CT slices obtained at the midpoint along the length of each fragment in the direction where the osteon cross-section could be observed. Osteon and Haversian canal contours were traced, and areas were calculated using ImageJ (National Institutes of Health, USA). All histomorphometrical data of the Fukui Cave specimens were collected by one of the authors (J.S.).

### Comparative mammal bones

For comparative reference, histological sections of compact bone were taken from the midshaft of limb bones (humerus, radius, femur, and tibia) or the mandibular body of the following taxa: Naumann’s elephant (*Palaeoloxodon cf. naumanni*), unidentified Pleistocene elephant fossil (Elephantidae), Asian elephant (*Elephas maximus*), human (*Homo sapiens*), macaque (*Macaca fuscata*), leporid (Leporidae), wild boar (*Sus scrofa*), sika deer (*Cervus nippon*), ancient sika deer (Cervus praenipponicus), reindeer (Rangifer tarandus), Japanese serow (*Capricornis crispus*), Yabe’s giant deer (Sinomegaceros yabei), cattle (*Bos taurus*), bison (*Bison bison*), Japanese marten (*Martes melampus*), dog (*Canis lupus*), raccoon dog (*Nyctereutes procyonoides*), red fox (*Vulpes vulpes*), leopard (*Panthera pardus*), and brown bear (*Ursus arctos*). These animals represent terrestrial mammals that inhabited the Japanese Archipelago during the Late Pleistocene or Holocene^31–33,97^. Detailed information on specimen IDs, sample numbers, and attributes of the comparative materials is provided in Table 6.

**Table 6:** Comparative specimens.

Taxon	Specimen ID	Skeletal element	*N*.B	Data source
Proboscidea				
Naumann’s elephant (*Palaeoloxodon* cf. *naumanni*)	PLN01Tp	Tib ant mid	1	This study (On), Sawada et al. 2014 (H)
Pleistocene elephant fossil (Elephantidae)	EKZ97Fa	Fem ant mid	1	This study (On), Sawada et al. 2014 (H)
Asian elephant (*Elephas maximus*)	Ele01Fp	Fem ant mid	1	This study (On), Sawada et al. 2014 (H)
Primates				
Human (*Homo sapiens*)	Homo01Ha	Hum ant mid	1	Sawada et al. 2010
Human (*Homo sapiens*)	Homo03Ha	Hum ant mid	1	Sawada et al. 2010
Human (*Homo sapiens*)	Homo01Fa	Fem ant mid	1	Sawada et al. 2010
Human (*Homo sapiens*)	Homo02Fa	Fem ant mid	1	Sawada et al. 2010
Human (*Homo sapiens*)	Homo04Fa	Fem ant mid	1	Sawada et al. 2010
Human (*Homo sapiens*)	Homo05Fa	Fem ant mid	1	Sawada et al. 2010
Human (*Homo sapiens*)	Homo06Fa	Fem ant mid	1	Sawada et al. 2010
Human (*Homo sapiens*)	HomoPf01	Fem ant mid	15	Pfeiffer et al. 2006
Human (*Homo sapiens*)	HomoPf02	Fem ant mid	20	Pfeiffer et al. 2006
Human (*Homo sapiens*)	HomoPf03	Fem ant mid	20	Pfeiffer et al. 2006
Macaque (*Macaca fuscata*)	Mac01Fa	Fem ant mid	1	This study
Macaque (*Macaca mulatta*)	MacHa01	Fem mid	41	Havill 2004
Macaque (*Macaca mulatta*)	MacHa02	Fem mid	34	Havill 2004
Lagomorpha				
Leporid (Leporidae)	Lep01Hp	Hum post mid	1	This study
Leporid (Leporidae)	Lep01Fl	Fem lat mid	1	This study
Rabbit (*Oryctolagus cuniculus*)	RabMa	Fem mid	10	Martiniaková et al. 2007
Medium-sized Artiodactyla				
Wild boar (*Sus scrofa*)	Sus01Ha	Hum ant mid	1	Sawada et al. 2010
Wild boar (*Sus scrofa*)	Sus02Ha	Hum ant mid	1	Sawada et al. 2010
Wild boar (*Sus scrofa*)	Sus01Fa	Fem ant mid	1	Sawada et al. 2010
Wild boar (*Sus scrofa*)	Sus02Fa	Fem ant mid	1	Sawada et al. 2010
Sika deer (*Cervus nippon*)	Cer02Ha	Hum ant mid	1	This study (On), Sawada et al. 2014 (H)
Sika deer (*Cervus nippon*)	Cer03Ha	Hum ant mid	1	This study (On), Sawada et al. 2014 (H)
Sika deer (*Cervus nippon*)	Cer01Ra	Rad ant mid	1	This study (On), Sawada et al. 2014 (H)
Sika deer (*Cervus nippon*)	Cer01Fa	Fem ant mid	1	This study (On), Sawada et al. 2014 (H)
Sika deer (*Cervus nippon*)	Cer05Fa	Fem ant mid	1	This study (On), Sawada et al. 2014 (H)
Sika deer (*Cervus nippon*)	Cer01Fp	Fem post mid	1	Sawada et al. 2010
Sika deer (*Cervus nippon*)	Cer02Fp	Fem post mid	1	Sawada et al. 2010
Sika deer (*Cervus nippon*)	Cer03Fp	Fem post mid	1	Sawada et al. 2010
Sika deer (*Cervus nippon*)	Cer01Ta	Tib ant mid	1	This study (On), Sawada et al. 2014 (H)
Sika deer (*Cervus nippon*)	Cer04Ta	Tib ant mid	1	This study (On), Sawada et al. 2014 (H)
Ancient sika deer (*Cervus praenipponicus*)	CPR91Fp	Fem post mid	1	This study (On), Sawada et al. 2014 (H)
Ancient sika deer (*Cervus praenipponicus*)	CPR92Ta	Tib ant mid	1	This study (On), Sawada et al. 2014 (H)
Reindeer (*Rangifer tarandus*)	Ran01Fp	Fem post mid	1	This study
Japanese serow (*Capricornis crispus*)	Cap01Fa	Fem ant mid	1	Sawada et al. 2010
Large-sized Artiodactyla				
Yabe’s giant deer (*Sinomegaceros yabei*)	SMY01M	Mand bas	1	This study (On), Sawada et al. 2014 (H)
Yabe’s giant deer (*Sinomegaceros* cf. *yabei*)	SMY02Ra	Rad ant mid	1	This study (On), Sawada et al. 2014 (H)
Cattle (*Bos taurus*)	Bos01Ha	Hum ant mid	1	This study (On), Sawada et al. 2014 (H)
Cattle (*Bos taurus*)	Bos04Ha	Hum ant mid	1	Sawada et al. 2010
Cattle (*Bos taurus*)	Bos05Ha	Hum ant mid	1	This study (On), Sawada et al. 2014 (H)
Cattle (*Bos taurus*)	Bos01Ra	Rad ant mid	1	This study (On), Sawada et al. 2014 (H)
Cattle (*Bos taurus*)	Bos02Fa	Fem ant mid	1	Sawada et al. 2010
Cattle (*Bos taurus*)	Bos03Fa	Fem ant mid	1	Sawada et al. 2010
Cattle (*Bos taurus*)	BosMa	Fem mid	8	Martiniaková et al. 2007
Bison (*Bison bison*)	Bis01Fp	Fem post mid	1	This study (On), Sawada et al. 2014 (H)
Medium-sized Carnivora				
Japanese marten (*Martes melampus*)	Mar01Ha	Hum ant mid	1	This study (On), Sawada et al. 2014 (H)
Dog (*Canis lupus*)	Can01Fa	Fem ant mid	1	This study (On), Sawada et al. 2014 (H)
Dog (*Canis lupus*)	Can01Ta	Tib ant mid	1	This study (On), Sawada et al. 2014 (H)
Raccoon dog (*Nyctereutes procyonoides*)	Nyc01Fa	Fem ant mid	1	This study (On), Sawada et al. 2014 (H)
Raccoon dog (*Nyctereutes procyonoides*)	Nyc01Fp	Fem post mid	1	This study (On), Sawada et al. 2014 (H)
Red fox (*Vulpes vulpes*)	Vul01M	Mand bas	1	This study (On), Sawada et al. 2014 (H)
Leopard (*Panthera pardus*)	Pan01Fa	Fem ant mid	1	This study (On), Sawada et al. 2014 (H)
Large-sized Carnivora				
Brown bear (*Ursus arctos*)	Urs01Ha	Hum ant mid	1	Sawada et al. 2010
Brown bear (*Ursus arctos*)	Urs01Ra	Rad ant mid	1	This study (On), Sawada et al. 2014 (H)
Brown bear (*Ursus arctos*)	Urs01Fa	Fem ant mid	1	Sawada et al. 2010
Brown bear (*Ursus arctos*)	Urs01Ta	Tib ant mid	1	This study (On), Sawada et al. 2014 (H)

Skeletal element: Hum = Humerus; Rad = Radius; Fem = Femur; Tib = Tibia; Mand = Mandible; ant = anterior, post = posterior, lat = lateral, mid = midshaft; bas = basal cortex. N.B = Number of bones. Data source: On = On.Ar; H = H.Ar. All specimens for which the data source is indicated as ‘This study’ are curated at the Institute of Physical Anthropology, Niigata University of Health and Welfare.

Although no Late Pleistocene mammalian remains have been reported from the vicinity of Fukui Cave, fossils of Naumann’s elephant, macaque, ancient sika deer, and Yabe’s giant deer have been recovered from presumed Late Pleistocene deposits in limestone caves in the Hiraodai area, approximately 120 km from the site^98^. In addition, wild boar remains have been identified from the earliest Holocene layers of Fukui Cave^99^. As noted in the Introduction section, the excavated bone fragments were considered to originate from terrestrial mammals larger than small animals; therefore, small mammals such as rodents, moles, bats, and weasels were excluded from comparison.

For the comparative animal specimens, histological sections of compact bone were examined under transmitted and polarized light microscopy to record microstructural features. Micrographs were captured using a light microscope (Axio Imager.A1, Zeiss) equipped with a CMOS camera (AdvanCam-CTR20, AdvanVision). The cross-sectional areas of complete osteons and their Haversian canals were measured with ImageJ, following the procedures of Pfeiffer^67^ and Sawada et al.^18,69^. For some comparative animals, osteon and Haversian canal area data reported by Habill^100^, Martiniaková et al.^65^, Pfeiffer et al.^68^, and Sawada et al.^18,101^ were also used (Table 5).

Optical microscopy was used to observe histological structures in the comparative animal specimens instead of the CT imaging applied to the burned bones from Fukui Cave. Ideally, the same CT imaging should also have been applied to the comparative animal specimens; however, synchrotron-based CT image analysis requires substantial resources and limited access, making such validation difficult. Although CT and optical microscopy differ in image contrast and spatial resolution, the structures targeted in bone histomorphometric analysis (osteons and Haversian canals) are morphological features that can be observed using both methods. A previous study comparing histomorphometric measurements, including Haversian canal diameter, between µCT images of compact bone and microscopic images of histological sections reported no significant differences between the two methods^102^, supporting the validity of using optical microscopic images for comparison in the present study.

### Histomorphological shrinkage of bone specimens due to burning

Calcination causes the loss of organic components, an increase in the crystallinity of bioapatite, and a reorganization of bone structure^7,10,13,103,104^. Therefore, the effects of these physicochemical changes must be taken into account when performing bone morphometric analysis.

It is well known that bones shrink when exposed to high temperatures^105–107^, although the degree of shrinkage varies depending on the burning temperature, duration, and the state of the bone during burning^55,108^. As described in the Materials section, the burned bone fragments from Fukui Cave exhibited a gray color, suggesting they were burned at approximately 500–700 °C.

Regarding the shrinkage of osteons and Haversian canals due to burning, Absolonová et al.^96^ measured the cross-sectional areas of osteons and Haversian canals in both unburned and burned bones, providing detailed data. Their results indicate that bones burned at 500 °C and 600 °C showed no shrinkage of osteons or Haversian canals. In bones burned at 700 °C, however, the osteon area decreased to 76.6% of the pre-burning value (mean osteon area before burning: 28,089.85 μm²; mean osteon area after burning at 700 °C: 21,504.71 μm²), and the Haversian canal area decreased to 76.4% (mean Haversian canal area before burning: 2,172.26 μm²; mean after burning: 1,659.02 μm²).

Based on this experimental results, our study assumed that the On.Ar and H.Ar of the burned bone fragments from Fukui Cave fell within a range defined by: (1) no change due to burning, i.e., the measured values equal the original values (corresponding to 500–600 °C), and (2) shrinkage to 75% of the original values (corresponding to approximately 700 °C). The upper bound of the burning temperature was set at 700 °C, which seems reasonable considering that Hanson and Cain^8^ noted the histological structure becomes indistinct in bones fired at higher temperatures, and Squires et al.^95^ reported that microstructural identification becomes difficult in bones exposed to temperatures above 900 °C.

The lower and upper bounds of On.Ar and H.Ar for the estimated burning temperature range were calculated using the following formulas:

**(1) Lower bounds** (assuming a burning temperature of 500–600 °C): no shrinkage, so the measured value equals the original value.

On.Ar (or H.Ar) before burning = actual measured value of On.Ar (or H.Ar).

**(2) Upper bounds** (assuming a burning temperature of 700 °C ): shrinkage due to burning, so the measured value equals 75% of the original value.

On.Ar (or H.Ar) before burning = actual measured value of On.Ar (or H.Ar) / 0.75.

### Statistical analysis

For both On.Ar and H.Ar, differences in mean values were tested between the burned bone fragments from Fukui Cave and the comparative animal specimens. For the comparative specimens, only the data measured in the present study and those reported by Sawada et al.^18^ were used. Considering the shrinkage caused by burning in the Fukui Cave fragments, two sets of comparisons were performed: one assuming no shrinkage and the other assuming the maximum estimated shrinkage due to burning. As the assumption of equal variances was not met for either On.Ar or H.Ar, the Games–Howell test was applied. The significance level was set at 0.05, and all statistical analyses were conducted using SPSS Statistics (IBM).

### Ethics statement

This study did not involve living humans or animals. The primary materials analyzed were archaeological faunal remains dating to approximately 16,000 years ago. Additionally, the comparative specimens used in this study were obtained from the permanent osteological collections of the Institute of Physical Anthropology, Niigata University of Health and Welfare. All specimens were used in accordance with the institutional guidelines for the use of archival biological materials. No animals were specifically sacrificed for this research.

## Data Availability

All data supporting the findings of this study are available at Zenodo: https://zenodo.org/records/17688116.
